# Risperidone-associated adverse drug reactions and *CYP2D6* polymorphisms in a South African cohort

**DOI:** 10.1016/j.atg.2015.05.001

**Published:** 2015-05-14

**Authors:** Tyren M. Dodgen, Arinda Eloff, Connie Mataboge, Louw (.J.L.). Roos, Werdie (.C.W.). van Staden, Michael S. Pepper

**Affiliations:** a Department of Pharmacology, School of Medicine, University of Pretoria, South Africa; b Department of Immunology, School of Medicine, University of Pretoria, South Africa; c Department of Psychiatry, School of Medicine, University of Pretoria, South Africa; d Institute for Cellular and Molecular Medicine, Faculty of Health Sciences, University of Pretoria, South Africa; e Department of Genetic Medicine and Development, Faculty of Medicine, University of Geneva, Geneva, Switzerland

**Keywords:** Risperidone, Extrapyramidal symptoms, Weight gain, CYP2D6

## Abstract

**Background:**

Contradictory information exists regarding the influence of *CYP2D6* polymorphisms on adverse drug reactions (ADRs) (extrapyramidal symptoms (EPS) and weight gain) related to risperidone treatment. This prompted us to evaluate the influence of *CYP2D6* genetic variation in a cohort of South African patients who presented with marked movement disorders and/or weight gain while on risperidone treatment.

**Methods:**

Patients who were experiencing marked risperidone ADRs were recruited from Weskoppies Public Psychiatric Hospital. As poor or intermediate metabolism was expected, comprehensive *CYP2D6* sequence variations were evaluated using XL-PCR + Sequencing.

**Results:**

No statistically significant association was found between *CYP2D6* poor metabolism and risperidone ADRs. An inverse relationship between EPS and weight gain was however identified. A novel *CYP2D6* allele was identified which is unlikely to affect metabolism based on *in silico* evaluation.

**Conclusion:**

*CYP2D6* variation appeared not to be a good pharmacogenetic marker for predicting risperidone-related ADRs in this naturalistic South African cohort. Evaluation of a larger cohort would be needed to confirm these observations, including an examination of the role of potential intermediaries between the hypothesised genetic and clinical phenotypes.

## 1 Introduction

Atypical antipsychotic medication has largely superseded typical or classical antipsychotics for the treatment of schizophrenia in the last two decades. This is largely due to improved efficacy in treating negative symptoms of schizophrenia as well as a more favourable side effect profile (less extrapyramidal symptoms and tardive dyskinesia). Demonstrating antagonism at 5-hydroxytryptamine (5-HT_2_) and dopamine (D_2_) receptors (Janssen et al., 1988, Leysen et al., 1988), risperidone is an atypical antipsychotic which improves both positive and negative symptoms of schizophrenia (Singam et al., 2011). Risperidone is also registered for the treatment of manic episodes of bipolar disorder and for irritability associated with autistic disorder. Off-label uses include behavioural problems in dementia, refractory or psychotic major depressive disorder, refractory obsessive–compulsive disorder and Tourette's disorder, among others (Shekelle et al., 2007). The range of patients receiving atypical antipsychotic treatment (including risperidone) for a variety of different indications is vast and includes children (Lazzeretti et al., 2011) as well as the elderly (Burke and Tariot, 2009).

Although the atypical antipsychotics have an improved side effect profile, adverse drug reactions (ADRs) still pose a challenge. Genetic polymorphisms and environmental influences are typically implicated in ADRs. A study of 500 French patients taking risperidone revealed that dosage adjustments were needed in 61% of patients due to the effects of co-medication and 10% as a result of genetic factors (Martin et al., 2004). Understanding the contribution of environmental factors, particularly drug–drug interactions resulting from polypharmacy (common in psychiatry treatment), and also of genetic influences, may assist in reducing ADRs. Environmental factors will persist as long as the factor is present, and can often be modified. However, genetic influences are permanent and need to be accounted for (de Leon et al., 2008).

One of the most important genetic factors influencing risperidone pharmacokinetics is phase I metabolism mediated predominantly by *CYP2D6* (Fleeman et al., 2011). *CYP2D6* is a highly polymorphic gene with over 100 alleles identified to date (http://www.cypalleles.ki.se/cyp2d6.htm). Many of these alleles have been found to alter enzyme function, ranging from absent to increased. Of these alleles, the non-functional CYP2D6*4 allele is particularly frequent in individuals of European origin (Sistonen et al., 2009) and *5 is found in the Coloured and Xhosa populations of South African (Gaedigk and Coetsee, 2008, Wright et al., 2010). Frequent decreased function alleles include CYP2D6*10 in East Asians, *17 and *29 in Black Africans and *41 in Europeans as well as in western and southern Asians (Sistonen et al., 2009). Multiple copies of functional *CYP2D6* genes are frequent in much of Africa (Alessandrini et al., 2013). The *CYP2D6* alleles cause inter-individual variations in metabolism which are classified as poor (PM), intermediate (IM), extensive (EM) and ultra-rapid (UM). This may form a vital tool for improving efficacy and reducing ADRs by predicting phenotype *i.e.* metabolism.

Weight gain is another important ADR of risperidone (Nasrallah, 2008). Weight gain results in reduced patient compliance irrespective of symptomatic improvement (Nasrallah, 2006). Although there is very little known about the association between *CYP2D6* polymorphisms and weight gain, Lane et al. (2006) observed a significant correlation between the *10 allele and weight gain in risperidone-treated patients.

In light of contradicting data on *CYP2D6* pharmacogenetics and risperidone ADRs (Fleeman et al., 2011), and given the great genetic diversity of southern African populations, this pilot study was aimed at addressing this relationship in a small cohort of South African patients. *CYP2D6* genotype was analysed in a risperidone treated cohort selected on the basis of risperidone ADRs, specifically movement disorders and weight gain. Notwithstanding intermediaries between the genetic phenotype of poor metabolisers and the clinical phenotype of ADRs, the aim of this pilot study was to examine whether a predicted *CYP2D6* PM phenotype would emerge in a naturalistic cohort for which such phenotype would be most likely, *i.e.* a cohort with clinically marked movement disorders and/or weight gain.

## 2 Materials and methods

### 2.1 Patient cohort

Approval was obtained from the Research Ethics Committee, Faculty of Health Sciences, University of Pretoria and the study was performed according to the stipulations of the Declaration of Helsinki. Written informed consent was obtained from all patients. Inpatients or outpatients at Weskoppies Public Psychiatric Hospital (Pretoria, South Africa) receiving risperidone treatment were recruited if they experienced a clinically marked risperidone related movement disorder and/or weight gain as had been recorded in their clinical notes before recruitment to the study. A cohort was thus purposively gathered for which a link between clinical phenotype (of marked ADRs) and genetic phenotype (of a PM nature) might be present. A naturalistic cohort of 24 patients older than 17 years of age, who gave informed consent, who were of any race and either gender, irrespective of their psychiatric diagnosis and irrespective of whether they used concomitant medication, was gathered, except for excluding patients concomitantly on an anti-psychotic medication other than risperidone. Patients suffering from neurological disorders that may be mistaken for risperidone related ADRs were excluded from the cohort. On the day of recruitment, one of the collaborating psychiatrists (MD level) performed clinical measures of the ADRs and drew two venous blood samples in ethylenediaminetetraacetic acid (EDTA) vacutainer tubes (Becton-Dickinson, Franklin Lakes, NJ, USA) for *CYP2D6* genotypic evaluation.

### 2.2 Phenotypic evaluation

ADRs resulting from risperidone treatment were measured as clinical phenotype in this study. Two distinct and prominent ADRs were measured: weight gain and movement disorders. If either prompted inclusion into the study, weight gained from the onset of risperidone treatment until the time of recruitment was recorded as a continuous variable. Dyskinesia (irrespective of whether acute or tardive) was measured using the Abnormal Involuntary Movement Scale (AIMS, Munetz and Benjamin, 1988), akathisia using the Barnes Akathisia Scale (BAS, Barnes, 1989) and Parkinsonism using the Simpson-Angus Scale (SAS, Simpson and Angus, 1970). No patients suffered from acute dystonia at the time of measurement.

### 2.3 CYP2D6 genotyping

Genomic DNA (gDNA) was extracted from whole blood using the automated Maxwell® 16 system (Promega, Madison, WI, USA) according to instructions. Genotyping was performed using a *CYP2D6* XL-PCR + Sequencing strategy as previously described (Dodgen et al., 2013). Briefly, the strategy makes use of two duplex long-range PCR (polymerase chain reaction) assays, one for CYP2D6*5 (complete gene deletion) and the other for duplication detection followed by *CYP2D6* gene sequencing for allele determination. *CYP2D6* gene sequencing was performed by Inqaba Biotechnological Industries (Pretoria, South Africa) using 3130XL and 3500XL (Applied Biosystems) instruments. PCR products were purified using FastAP Thermosensitive Alkaline Phosphatase (Fermentas Life Science) according to the manufacturer's instructions. The ABI Big Dye Terminator Cycle Sequencing kit version 3.1 (Applied Biosystems) and appropriate *CYP2D6* sequencing primers were used for sequencing reactions.

Resulting electropherograms were edited using FinchTV version 1.4.0 (Copyright © 2004–2006, Geospiza Inc.) and compared to the AY545216 (GenBank) in CLC DNA Workbench version 5.5 (CLC bio, Aarhus, Denmark) software for polymorphism identification. Single nucleotide polymorphisms (SNPs) were numbered according to M33388 (GenBank) and alleles identified according to the Human CYP Nomenclature website for *CYP2D6* (http://www.cypalleles.ki.se/index.htm).

The novel polymorphism was cloned using the CloneJET™ PCR Cloning Kit (Fermentas Life Science) according to the manufacturer's instructions and transformed into DH5α cells (Zymo Research, Orange, CA, USA). Colonies were screened by amplifying the region of interest (where the novel SNP was located). Once the novel SNP was found the clone was amplified and re-sequenced. The novel SNP was evaluated using *in silico* software, Sorting Intolerant from Tolerant (SIFT) and PolyPhen prediction software to estimate the potential effect on CYP2D6 activity (Ramensky et al., 2002, Ng and Henikoff, 2003). The novel allele has been submitted to the Human CYP Nomenclature committee for *CYP2D6* allele designation.

### 2.4 CYP2D6 enzyme activity prediction

An adjusted version of the Activity Score (AS) system (Gaedigk et al., 2008) was used to predict CYP2D6 phenotype. The adjusted phenotype prediction system was adopted from genotype–phenotype comparisons described by Dodgen et al. (2013), where activity is Increased = 2.0, Normal = 1.0, Decreased = 0.5 and None = 0.0 using information from the Human Cytochrome P450 (*CYP*) Allele Nomenclature Committee's online database for *CYP2D6*. CYP2D6*17, traditionally assigned an AS of 0.5, was given a score of 1.0, as this allele has been found to metabolise risperidone with full function (de Leon et al., 2009). If no information on phenotypic activity was available, the allele was assigned a score of 1.0. Summation of genotypic scores (e.g.*2 / *41 = 1.0 + 0.5 = 1.5) predicted phenotype as PM = 0.5, IM = 0.5–1.0, EM = 1.5–2.0 and UM > 2.0.

### 2.5 Statistical analyses

Tools for Population Genetic Analysis (TFPGA) software v1.3 was used to test allele deviation from Hardy–Weinberg equilibrium using Fisher's exact test (Miller, 1997). Additional statistical evaluations were calculated using SPSS version 20.0 (SPSS Inc., Chicago, Ill). As a normal distribution of data could not be assumed, nonparametric tests were used for comparison. The Mann–Whitney test was used following cross tabulation to compare sex and race (Black African or White Caucasian) to ADRs. The Kruskal–Wallis test, using the Chi-square, was used to compare predicted phenotype with each risperidone movement disorder experienced as well as weight gained (ADRs). Kendall's tau-b was used to compare age, number of cigarettes smoked, risperidone dosage, and each ADR to the other. Concomitant medication, anticholinergic medication, sex and race were evaluated as confounding influences of risperidone ADRs using the Mann–Whitney test. *P* values of < 0.05 were considered significant.

## 3 Results

### 3.1 Patient characteristics

The observed characteristics for the cohort of 24 risperidone-treated patients experiencing movement disorders and/or weight gain are presented in Table 1. Parkinsonism (SAS, *n* = 18) appeared to be the most commonly experienced ADR followed closely by dyskinesia (AIMS, *n* = 17). Eight of the patients were included by virtue of their clinically marked weight gain, of which two also experienced simultaneous movement disorders, but in both these cases the weight gain was less than 5 kg.

None of the patients admitted to the use of cannabis at the time of sampling. Eight patients were prescribed risperidone monotherapy. Concomitantly prescribed anticholinergic medication included orphenadrine (*n* = 5) and biperidine (*n* = 3). Additional concomitant medication at the time of sampling included sodium valproate (*n* = 8), lithium (*n* = 4), oxazepam (*n* = 4), clonazepam (*n* = 3), cyproteroneacetate (*n* = 2), fluoxetine (*n* = 2), propranolol (*n* = 2), thyroxin (*n* = 2), venlafaxine hydrochloride (*n* = 2), carbamazepine (*n* = 1), citalopram (*n* = 1), hydroxyzine (*n* = 1), imipramine (*n* = 1), metformin (*n* = 1), omeprazole (*n* = 1), paroxetine (*n* = 1) and perindopril (*n* = 1).

### 3.2 CYP2D6 genotype and predicted phenotype

All *CYP2D6* alleles identified were in Hardy–Weinberg equilibrium. Of the 12 different *CYP2D6* alleles identified (Table 2), three were responsible for absent enzyme function (*4, *5 and *6B), four were responsible for reduced enzyme function (*10B, *17, *29 and *41) and one novel allele was identified. No duplications (functional or non-functional) were identified. Table 2 also shows the genotypes observed, the AS scores and predicted phenotype. PM was predicted in one patient (4.2%), 12 were IM (50.0%) and 11 were EM (45.8%). There were no UM's predicted in this cohort.

### 3.3 Novel allele

**Fig. 1:**
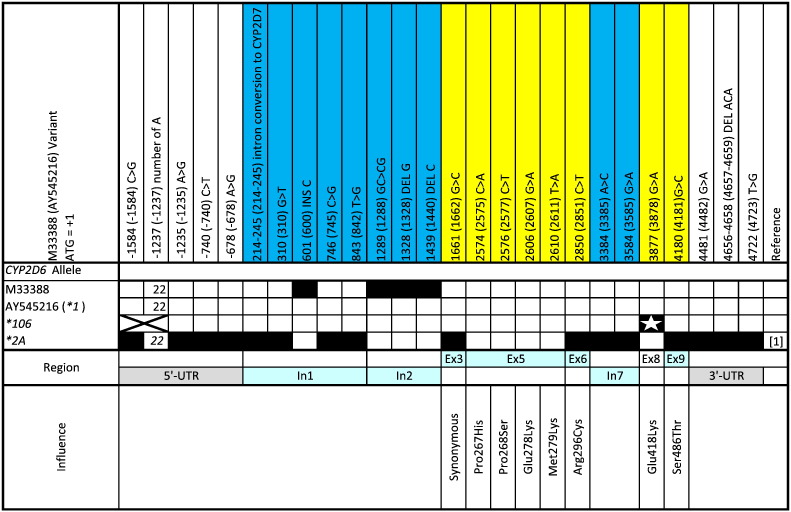
Comparison of the novel *CYP2D6* alleles identified in this cohort to similar alleles. M33388 and AY545216 reference sequences were used for numbering and as CYP2D6*1 respectively. Black boxes represent sequence variation at a specific locus. A star in a black box represents a novel SNP resulting in non-synonymous amino acid changes and thus allele defining (or sub-variant defining) SNPs, and an x represents regions not sequenced. M33388, AY545216 and CYP2D6*2A are depicted as presented on the Nomenclature Committee website (www.cypalleles.ki.se) or as represented by Gaedigk et al. (2007).

Fig. 1 illustrates the novel allele identified in this cohort. The non-synonymous 3877G > A SNP was identified in exon 8 with a *1 backbone. This SNP resulted in amino acid change E418K. No functional difference was found by *in silico* PolyPhen software, predicting the amino acid change to be benign with a PSIC score of 1.178. A second *in silico* prediction by SIFT software agreed with PolyPhen predicting that this mutation would be tolerated with a SIFT score of 0.13.

### 3.4 Risperidone ADRs and CYP2D6 influence

None of the individual risperidone-associated movement disorders were found simultaneously, as correlation coefficients were poor and *P* values were all above 0.23 (refer to Table 1). This suggests that dyskinesia, akathisia and parkinsonian symptoms experienced as a result of risperidone therapy are independent of one another. A strong negative correlation of − 0.484 was found between weight gain and akathisia (*P* = 0.004) and − 0.435 between weight gain and parkinsonian symptoms (*P* = 0.010). This suggests that when weight gain is experienced, the patient is unlikely to experience akathesia or parkinsonism and *vice versa*. Tardive diskinesia had a weak correlation of − 0.136 with weight gain (*P* = 0.458), and therefore may not share the same relationship with weight gain as the other risperidone movement disorders. When *CYP2D6* predicted phenotype was compared to risperidone ADRs, no statistical association was found, with *P* values ≥ 0.335 (Table 1).

### 3.5 Confounding variables which may be associated with risperidone ADRs

Neither concomitant medication (*P* ≥ 0.081) nor the number of cigarettes smoked (*P* ≥ 0.124; Table 1) were found to influence risperidone ADRs. Age, race, gender and sex appeared not to be associated with risperidone ADRs with *P* values ≥ 0.086 (refer to Table 1). Sex correlated well with symptoms of parkinsonism (*P* = 0.038), suggesting that women are more likely to experience parkinsonism than men when treated with risperidone. Although not statistically significant (*P* = 0.086), the Black African portion of the cohort was apparently more susceptible to dyskinesia. The latter two comparisons need to be interpreted with caution as the sample size in this cohort was small and a larger cohort will need to be sampled to confirm the association.

## 4 Discussion

### 4.1 CYP2D6 genetics

As all alleles were in Hardy–Weinberg equilibrium, the *CYP2D6* screening assay using XL-PCR + Sequencing appears to be accurate and comprehensive for *CYP2D6* allele identification in this cohort. CYP2D6*4, *6B (absent function) and *41 (reduced function) were observed in Caucasians, while *17 and *29 (both reduced function) were observed in Black Africans. One Black African individual was heterozygous for CYP2D6*4, an allele which is observed at low frequency in African populations (Sistonen et al., 2009). CYP2D6*10B (reduced function) has been found at high frequency in African populations (Matimba et al., 2009). It is therefore not surprising to find CYP2D6*10B in Black Africans in this cohort. CYP2D6*5 was found in both Black Africans and Caucasians which agrees with what has been observed in cohorts of varying ethnicity (Sistonen et al., 2009). The novel allele identified was observed in a Black African individual, emphasising the importance of comprehensive genetic screening methods to accommodate low frequency alleles which may be of clinical relevance (Matimba et al., 2009). Confidence in the genotyping assay used, including copy number discrepancies, was validated by Dodgen et al. (2013) in which potential for bias or analytical error was addressed. As the sample size of each ethnic groups is small, allele frequencies will not be considered in this pilot cohort.

Although the novel allele was evaluated using *in silico* evaluation of change in enzyme activity, further *in vitro* or *in vivo* experiments will need to be conducted for confirmation.

### 4.2 CYP2D6 and risperidone movement disorders

The sampled cohort was selected for having marked risperidone-related ADRs (risperidone induced movement disorders and weight gain), Although the significance of *CYP2D6* polymorphisms affecting risperidone metabolism in both drug naïve and experienced patients has been well established (Jovanovic et al., 2010), it is still unclear whether genetic mutations in *CYP2D6* are associated with ADRs. If the link between reduced risperidone metabolism by CYP2D6 PMs and movement disorders was as clear as has previously been described (de Leon et al., 2005a, Bozina et al., 2008), the majority of this cohort would have been expected to be PMs. This was not the case, as only 4.2% (1/24) were PMs. This frequency for PMs has been similarly observed in various volunteer based southern African cohorts (Matimba et al., 2009, Wright et al., 2010, Dodgen et al., 2013). Half of the cohort was IMs and this should be investigated further as a potential explanation for ADRs.

Little research has been published on the association between risperidone induced movement disorders and *CYP2D6* polymorphisms and contradictory findings are apparent. The majority find no support for the association (Jovanovic et al., 2010), agreeing with what was observed in this study. This lack of association could be similar to the observed poor correlation between *CYP2D6* polymorphisms, risperidone active moiety and clinical response measured as prolactin levels released due to dopamine receptor occupation (Wang et al., 2007).

Although the plasma concentration of risperidone has been found to be higher in PM's and 9-hydroxyrisperidone is higher in EM's, the total active moiety (the sum of both) appears not to vary much between metaboliser classes (Novalbos et al., 2010). It has therefore been proposed that CYP2D6 metabolism is unlikely to be of clinical relevance in terms of efficacy.

Conflicting results have also been reported, where increased risperidone active moiety was found in *CYP2D6* predicted PMs compared to EMs (Locatelli et al., 2010). This, combined with the suggestion by de Leon et al. (2008), that risperidone and 9-hydroxyrisperidone are not equipotent (risperidone having greater activity), may in part explain the ADRs experienced by PMs *versus* EMs. In this case, ADRs may be due to the reduced rate of elimination of the accumulated active moiety in plasma seen with repeated dosing in PMs. The extended plasma half-life for risperidone in *CYP2D6* PMs from 2.9 h in EM to 15.1 h in PMs (Novalbos et al., 2010) combined with a typical dosage of 3.0 to 8.0 mg taken at two time intervals daily, could result in increasing plasma levels towards toxicity. In support of this, Kang et al. (2009) demonstrated that *CYP3A5* polymorphisms are more likely to influence the risperidone active moiety. Alternatively, both higher plasma concentrations of the active moiety (Locatelli et al., 2010) and the *CYP2D6* PM genotype (de Leon et al., 2005a) have been associated with an increase in the incidence of extrapyramidal symptom (EPS) ADRs.

### 4.3 CYP2D6 and risperidone weight gain

An important risperidone related ADR that has received little pharmacogenetic attention is weight gain. Weight gain has been found to reduce compliance in patients taking antipsychotics even if the psychopharmacological treatment is effective (Nasrallah, 2006). The CYP2D6*10 polymorphism 188C > T was found to be associated with weight gain in risperidone-treated Chinese patients (Lane et al., 2006). Interestingly, the association appeared to be stronger in heterozygote individuals (C/T) than in homozygotes (T/T). In the present study, *CYP2D6* defective polymorphisms were not associated with weight gain and only 1 of the 7 patients experiencing weight gain was a PM. In patients who gained more than 5 kg, risperidone-related movement disorders were not observed. This is an interesting observation, which may have a genetic component.

Factors unrelated to risperidone treatment or genetic predisposition may also have influenced weight gain. Such factors may include comfort/social eating, contraceptives, taste changes, changes in diet and the administered medication itself.

### 4.4 Inverse relationship between movement disorders and weight gain

The statistical observation of an inverse relationship between movement disorders and weight gain should be met with caution. Clinically, movement disorders tend to present early in treatment and are treated or overcome by dosage titration. Weight gain typically has a slow onset (Hellings et al., 2001) and the patients who have experienced risperidone-related weight gain have overcome the initial risperidone-related ADRs.

### 4.5 Concomitant medication

Many of the patients sampled in this naturalistic cohort were taking concomitant medication which may have confounded the ADRs observed. The P450 Drug Interaction Table posted by the Division of Clinical Pharmacology at Indiana University offers valuable information regarding drug substrates, inducers and inhibitors of the CYP enzymes (Flockhart, 2009). Applying this information to the current cohort allows one to evaluate the effect of concomitant medication. Citalopram, fluoxetine, imipramine, paroxetine and propranolol were prescribed concomitantly to some of the patients in this cohort and are listed as substrates of CYP2D6 (Table 1). More importantly, fluoxetine (Brynne et al., 1999, Flockhart, 2009) and paroxetine (Bertelsen et al., 2003, Flockhart, 2009) were listed as strong inhibitors of CYP2D6 causing more than a 5-fold increase in the plasma area under the curve (AUC) concentration and/or an 80% decrease in clearance of CYP2D6 substrates (Flockhart, 2009). It is therefore recommended that these medications should not be co-administered with risperidone, although sometimes this is unavoidable.

Three patients were concomitantly prescribed one of these drugs with risperidone. The first patient was an EM on fluoxetine, and received 4 mg risperidone per day and had a 15/40 score for SAS. The second patient was a PM on fluoxetine who received 2 mg risperidone per day, and scored 9/40 for AIMS and 14/40 for SAS. The third patient was an EM on paroxetine who received 6 mg risperidone daily, and scored 4/14 for BAS, 1/40 for SAS and gained 4 kg. These patients, in theory, should have experienced the worst risperidone-induced movement disorders, but this was not the case. Similarly, a different patient was co-prescribed hydroxyzine, which is listed as an inhibitor of CYP2D6 (Hamelin et al., 1998). This patient received 1 mg risperidone per day, was an EM and had higher degree of movement disorders than the previous three patients, scoring 21/40 for AIMS, 9/14 for BAS and 4/40 for SAS. In this case it would appear that hydrazine could have been contributing to perceived risperidone ADRs, but this was the only example in the cohort. Finally citalopram, listed as an inhibitor of CYP2D6, which was discounted previously due to lack of supporting documentation (Mannheimer et al., 2008), did not appear in this study to increase movement disorders when co-prescribed with risperidone, although this should be investigated in a larger cohort. The majority of the patients who gained more than 5 kg while on risperidone were not receiving concomitant medication, except one patient who gained 45 kg and who was co-medicated with citalopram.

### 4.6 Additional genes to consider

Other genes that affect risperidone efficacy and cause ADRs have previously been considered. Genes affecting metabolism (enzymes), drug or dopamine clearance as well as drug receptor variability could be responsible for risperidone movement disorders and may be important as pharmacogenetic markers. For example, *CYP3A5* (Kang et al., 2009) and *ABCB1* (Gunes et al., 2008) have been shown to influence the risperidone active moiety. Genetic variability of phase II metabolism by a glutathione S-transferase enzyme coded for by *GSTM1* has been associated with dyskinesia experienced in risperidone-treated patients (de Leon et al., 2005b). The Ser9Gly mutation in the *DRD3* dopamine receptor gene is associated with increased risk of dyskinesia in risperidone treated patients (de Leon et al., 2005b). Weight gain as a result of risperidone treatment has been linked to polymorphisms in 5-HT2A, 5-HT2C, 5-HT6 and BDNF (Lane et al., 2006).

## 5 Limitations

We are aware that caution needs to be exercised when interpreting these results as the numbers are relatively small and there are confounding factors that have been included in this naturalistic cohort. Although the aim of this study was to identify PMs from patients experiencing ADRs, the sample size may not be sufficient to have identified such relationship. In some cases large cohorts are needed to find significant association (de Leon et al., 2005b). Perhaps if the cohort size is increased an association will be found, but the question is whether this will be a strong pharmacogenetic marker to reduce ADRs, particularly in view of the important environmental influences including concomitant medication. Although no statistical evidence was found, concomitant medication may have confounded the results in our study and this will need to be considered in future studies of larger cohort size. Measuring blood concentrations of risperidone and its active metabolite in future studies may help to clarify the role of potential intermediaries in the hypothesised connection between genetic and clinical phenotypes.

## 6 Conclusion

*CYP2D6* polymorphisms appeared not to associate with risperidone ADRs (movement disorders and weight gain) in this pilot cohort of risperidone-treated South African patients. Weight gain and movement disorders appeared not to be experienced simultaneously when patients were treated with risperidone, but this might have been a peculiarity of the cohort. A novel *CYP2D6* mutation, which may have clinical relevance, was identified in this cohort. A larger cohort would be valuable to confirm the results of this study.

## Competing interests

The authors declare that they have no competing interests.

## Authors' contributions

TMD carried out the molecular analysis and drafted the manuscript. AE assisted with the sequencing. CM saw the patients and collected patient data. JLR and CWvS saw the patients and collected patient data, and participated in the design and coordination of the study. CWvS and MSP edited and finalised the manuscript. MSP conceived the study, was responsible for its overall coordination and raised the funding. All authors read and approved the final manuscript.

## Figures and Tables

**Table 1:** Descriptive statistical data for a pilot cohort of risperidone-treated South African patients (*n* = 24) experiencing ADRs, and evaluation of related and confounding factors.

	Descriptive statistics				Comparative statistics *P*-value (correlation coefficient if applicable)			
Number	Mean	SD	Range	AIMS	BAS	SAS	WG
Female/male	8/16				0.637	0.182	0.038	0.928
Black/white	9/15				0.086	0.656	0.694	0.227
Age		32.9	12.4	18–61	0.331 (0.150)	0.709 (− 0.062)	0.289 (0.161)	0.699 (0.063)
Cigarettes (per day)	11^a^	9.0	9.1	0–20	0.124 (− 0.257)	0.670 (0.077)	0.175 (− 0.224)	0.259 (0.200)
Dosage (mg/day)		3.9	1.8	1–7	0.714 (− 0.060)	0.703 (− 0.067)	0.642 (0.074)	0.903 (0.021)

*Adverse drug reactions (ADRs)*								
AIMS (max = 40)		8.0	7.6	0–22	–	0.291 (0.182)	0.233 (0.189)	0.004 (− 0.484)
BAS (max = 14)		2.1	3.6	0–11		–	0.752 (0.054)	0.458 (− 0.136)
SAS (max = 40)		7.2	6.7	0–20			–	0.010 (− 0.435)
Weight gained (WG)		6.9	13.3	0–45				–

*CYP2D6 predicted phenotype*								
PM	4				0.841	0.797	0.335	0.855
IM	9							
EM	11							

a More than 10 cigarettes per day. ADRs were compared to sex and race using the Mann–Whitney test. ADR occurrences were evaluated for correlation with other ADRs, age, cigarettes smoked and dosage using Kendall's tau-b. The Kruskal–Wallis test was used to evaluate whether predicted phenotypes significantly influence each ADR. This test does not identify where the difference is, but whether or not there is a statistical difference at all. In this case a *P*-value is generated per ADR and not per predicted phenotype. Movement disorders measured using Abnormal Involuntary Movement Scale (AIMS), the Barnes Akathisia Scale (BAS) and the Simpson-Angus Scale (SAS).

**Table 2:** *CYP2D6* genotype and predicted phenotype frequencies in a cohort of South African risperidone treated patients experiencing ADRs.

Genotype	AS	Predicted phenotype	Number (*n* = 24)	Ethnicity
*1/*1	2.0	EM	1	White
*1/*17	1	Black
*1/*2	2	Black
*2/*106	1	Black
*2/*2	2	White
*2/*43	1	Black
*1/*29	1.5	1	Black
*2/*41	1	White
*35/*41	1	White
*1/*4	1.0	IM	5	4 W/1 B
*2/*4	1	White
*2/*6B	1	White
*4/*35	1	White
*5/*17	1	Black
*5/*41	0.5	1	White
*5/*10B*	2	Black
*4/*4	0.0	PM	1	White

ADRs, adverse drug reactions; AS, risperidone modified version of the activity score system (Gaedigk et al., 2008); EM, extensive metabolism; IM, intermediate metabolism; PM, poor metabolism.
